# Disruption of hippocampal neuronal circuit function depends upon behavioral state in the APP/PS1 mouse model of Alzheimer’s disease

**DOI:** 10.1038/s41598-022-25364-2

**Published:** 2022-12-05

**Authors:** Heng Zhou, Hanyan Li, Niket Gowravaram, Moqin Quan, Naila Kausar, Stephen N. Gomperts

**Affiliations:** 1 MassGeneral Institute for Neurodegenerative Disease, Department of Neurology, Massachusetts General Hospital, Charlestown, MA USA; 2 Jiangsu Key Laboratory of Brain Disease and Bioinformation, Research Center for Biochemistry and Molecular Biology, Xuzhou Medical University, Xuzhou, China

**Keywords:** Neural circuits, Neuronal physiology, Neurological disorders, Preclinical research, Ageing, Neurophysiology

## Abstract

The Alzheimer’s disease-associated peptide amyloid-beta (Aβ) has been associated with neuronal hyperactivity under anesthesia, but clinical trials of anticonvulsants or neural system suppressors have, so far, failed to improve symptoms in AD. Using simultaneous hippocampal calcium imaging and electrophysiology in freely moving mice expressing human Aβ, here we show that Aβ aggregates perturbed neural systems in a state-dependent fashion, driving neuronal hyperactivity in exploratory behavior and slow wave sleep (SWS), yet suppressing activity in quiet wakefulness (QW) and REM sleep. In exploratory behavior and REM sleep, Aβ impaired hippocampal theta–gamma phase-amplitude coupling and altered neuronal synchronization with theta. In SWS, Aβ reduced cortical slow oscillation (SO) power, the coordination of hippocampal sharp wave-ripples with both the SO and thalamocortical spindles, and the coordination of calcium transients with the sharp wave-ripple. Physostigmine improved Aβ-associated hyperactivity in exploratory behavior and hypoactivity in QW and expanded the range of gamma that coupled with theta phase, but exacerbated hypoactivity in exploratory behavior. Together, these findings show that the effects of Aβ alone on hippocampal circuit function are profoundly state dependent and suggest a reformulation of therapeutic strategies aimed at Aβ induced hyperexcitability.

## Introduction

Calcium imaging and electrophysiology studies universally suggest that deposits of amyloid-beta (Aβ), the primary component of plaques in Alzheimer’s disease (AD), cause neuronal hyperactivity^1–4^, but such studies have been acquired under anesthesia or immobility^2–4^. Aβ-associated changes in excitability have been posited to contribute to neuronal firing instability and integrated homeostasis network collapse, including in the hippocampus, a brain region targeted early in the course of AD^5^. However, clinical trials of anticonvulsants or of neural system suppressors have, so far, failed to improve cognitive symptoms in AD^6–9^.

Network oscillations in the hippocampus entrain neuronal activity across the sleep–wake cycle in the service of learning and memory^10^. In exploratory behavior, the hippocampal local field potential (LFP) is characterized by the robust theta oscillation that plays a critical role in memory encoding^10,11^. Hippocampal theta also arises in rapid eye movement (REM) sleep, while irregular activity and sharp wave ripple (SWR) events implicated in memory consolidation characterize quiet wakefulness (QW) and slow wave sleep (SWS)^12^. As Aβ levels also fluctuate across the sleep–wake cycle, maximal in awake behavior and low in SWS^13^, we posited that its effects on network function and connectivity would vary markedly across the brain states of the sleep–wake cycle.

To determine how Aβ deposits impact hippocampal dynamic calcium activity and LFP rhythms and their coordination across the distinct brain states that underlie memory encoding and consolidation, here we acquired large scale dynamic calcium imaging in hippocampal CA1 neurons with the head-mounted miniscope (Inscopix™) in concert with concomitant cortical and hippocampal LFP recordings across the sleep–wake cycle of aged APPswe/PS1dE9 (APP/PS1) transgenic mice, an AD model that recapitulates Aβ plaque formation and hippocampal-dependent memory impairments^14^.

## Results

### Aβ’s disruption of CA1 calcium dynamics depends upon behavioral state

**Figure 1:**
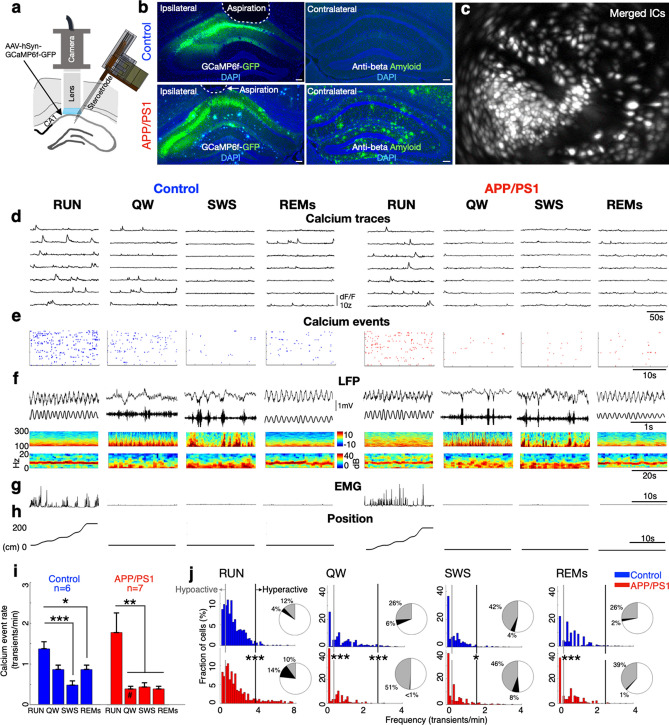
Aβ’s disruption of CA1 calcium dynamics depends upon behavioral state. (**a**) Schematic showing strategy for simultaneous dynamic calcium imaging and LFP recording in the hippocampus. (**b**) GCaMP6f and DAPI staining of control and APP/PS1 tissue. Scale bar 100 μm. (**c**) Identified CA1 neurons for calcium imaging in a single freely behaving mouse. (**d**) Calcium traces of imaged neurons across RUN, quiet wakefulness (QW), slow wave sleep (SWS) and rapid eye movement sleep (REMs) states. (**e**) Raster of calcium events across behavioral states (left, 130 neurons from a control mouse; right, 122 neurons from an APP/PS1 mouse). (**f**) LFP across states and genotypes. Hippocampal LFP (top, raw; bottom, in RUN and REMs, theta-filtered; bottom in QW and SWS, ripple-filtered). Spectrograms (top, hippocampus 100–300 Hz; bottom, in RUN and REM sleep, hippocampus 0–20 Hz; bottom, in QW and SWS, cortex 0–20 Hz). In RUN and REM sleep, the theta oscillation was prominent in s. radiatum (bottom spectrograms); in QW, sharp wave ripples (SWRs) were evident in the pyramidal cell layer (pcl) (top spectrogram, LFP traces). In SWS, SWRs in the hippocampal pcl (top spectrogram, LFP traces) and low frequency oscillations in cortex (bottom spectrogram) are evident. (**g**) EMG across states and genotypes. (**h**) The animal’s position on the linear track (RUN) and during the post-behavioral session (QW, SWS, and REM sleep) as a function of time. (**i**) In control mice, calcium event rates were higher in RUN behavior than in SWS and REM sleep (left); In APP/PS1 mice, calcium event rates in RUN behavior were higher than all other states (right). In direct comparisons, APP/PS1 mice had lower calcium event rates than control mice in QW (#). Mean calcium event rate in each mouse is shown. (**j**) APP/PS1-associated hyperactivity was increased in RUN and SWS, but reduced in QW and unchanged in REM sleep; APP/PS1-associated hypoactivity was evident in QW and REM sleep but not in RUN or SWS. All cells included. Grey: hypoactivity (< 0.25 transients/min); Black: hyperactivity (> 2 SD of mean calcium event rates in control mice). *^,#^*p* < 0.05; **p < 0.01; ***p < 0.001; ANOVA with post-hoc comparisons, 2-sided t-tests, χ^2^ tests. Control = 6 mice, 749 cells; APP/PS1 = 7 mice, 712 cells. Data in bar graphs are represented as mean ± S.E.M.

To evaluate hippocampal circuit function in a mouse model of Aβ-amyloidosis at an age when both Aβ plaque deposition (Fig. 1a, b) and hippocampal-dependent memory impairments are robust^14–18^, we applied somatic calcium imaging (Fig. 1c–e) and simultaneous ipsilateral LFP recording (Fig. 1f) in the hippocampal CA1 region of APP/PS1 transgenic mice (12.4 ± 1.2 months) and littermate controls (12.1 ± 2.3 months), as animals explored a linear track and subsequently rested in their home cage (Fig. 1g, h).

In control mice, calcium event rates were higher in exploratory behavior (RUN) than SWS and REM sleep (repeated measures ANOVA: F_(3,20)_ = 7.39, *p* = 0.0016, post hoc comparison: RUN vs. SWS, *p* = 0.0009; RUN vs. REM sleep, *p* = 0.028; Fig. 1i left), consistent with prior results^15^. However, this pattern was altered in APP/PS1 mice: Calcium event rates in RUN were significantly higher than all other states (repeated measures ANOVA: F_(3,24)_ = 6.94, *p* = 0.0016, post hoc comparison with RUN: quiet wakefulness (QW), *p* = 0.038; SWS, *p* = 0.0053; REM sleep, *p* = 0.0085; Fig. 1i right). In addition, compared to control, calcium event rates of APP/PS1 mice were reduced in QW (*p* = 0.0025, T = 3.89, *df* = 11; 2 sided t-test), but were comparable in other states (RUN *p* = 0.4, T = 0.7, *df* = 11; SWS *p* = 0.7, T = 0.3, *df* = 11; REM sleep *p* = 0.1, T = 1.6, *df* = 11; 2 sided t-tests; Fig. 1i).

The pattern of non-place cell event rates across behavioral states of APP/PS1 and control mice recapitulated these observations (control: repeated measures ANOVA: F_(3,20)_ = 3.81, *p* = 0.02, post hoc comparison: RUN vs. SWS, *p* = 0.0.01; APP/PS1: repeated measures ANOVA: F_(3,24)_ = 5.1, *p* = 0.007, post hoc comparison: RUN vs. QW, *p* = 0.01, RUN vs. SWS, *p* = 0.01, RUN vs. REM sleep, *p* = 0.02). In contrast, the pattern of place cell event rates across behavioral states was broadly similar in APP/PS1 mice and control mice (control: repeated measures ANOVA: F_(3, 20)_ = 18.2, *p* < 0.0001, post hoc comparison: RUN vs. QW, *p* = 0.0003, RUN vs. SWS, *p* < 0.0001, RUN vs. REM sleep, *p* = 0.0001; APP/PS1: repeated measures ANOVA: F_(3,24)_ = 13.7, *p* < 0.0001, post hoc comparison: RUN vs. QW, *p* = 0.0001, RUN vs. SWS, *p* = 0.0001, RUN vs. REM sleep, *p* = 0.0002; Supplementary Fig. S1). In addition, calcium activity of both place cells and non-place cells was reduced in QW in APP/PS1 compared to control mice (place cells: *p* = 0.007, T = 3.2, *df* = 11; non-place cells: *p* = 0.001, T = 4, *df* = 11; Supplementary Fig. S1).

To further evaluate the Aβ pathology induced change in excitation-inhibition balance, we first compared the distributions of activity in APP/PS1 and control mice. Within each state, distributions of calcium event rates differed across these genotypes (RUN, *p* < 0.0001; QW, *p* < 0.0001; SWS, *p* < 0.0001; REM sleep, *p* < 0.0001; Two-sample Kolmogorov–Smirnov (K–S) tests). We therefore evaluated the effect of Aβ pathology on dynamic calcium hypo- and hyperactivity within each state. In RUN, compared to control mice, APP/PS1 mice had a similar proportion of hypoactive cells (Chi = 1.7; *p* = 0.1; χ^2^ test) and a markedly increased proportion of hyperactive cells (Chi = 40.7, *p* < 0.0001; χ^2^ test). In QW, in contrast, APP/PS1 had a significantly increased proportion of hypoactive cells (Chi = 85.4, *p* < 0.0001; χ^2^ test) and a decreased proportion of hyperactive cells (Chi = 32.3, *p* < 0.0001; χ^2^ test). In SWS, APP/PS1 and control mice had a similar proportion of hypoactive cells (Chi = 0.4, *p* = 0.4; χ^2^ test) and an increased proportion of hyperactive cells (Chi = 5.8, *p* = 0.01; χ^2^ test). Finally, in REM sleep, APP/PS1 had an increased proportion of hypoactive cells compared to control mice (Chi = 22.3, *p* < 0.0001; χ^2^ test), with a similar proportion of hyperactive cells (Chi = 2.3, *p* = 0.1; χ^2^ test) (Fig. 1j). The proportion of aberrantly active cells that are place cells in each behavioral state is shown in Supplementary Table 1. The behavior of APP/PS1 and control animals was comparable during the imaging acquisitions (Supplementary Table 2). Together, these findings show that Aβ perturbs hippocampal calcium activity in a state-dependent manner, driving hyperactivity in RUN and SWS but suppressing activity in QW and REM sleep.

### Aβ impairs cortical–hippocampal coordination and modulation of hippocampal calcium activity at sharp wave ripples in slow wave sleep

**Figure 2:**
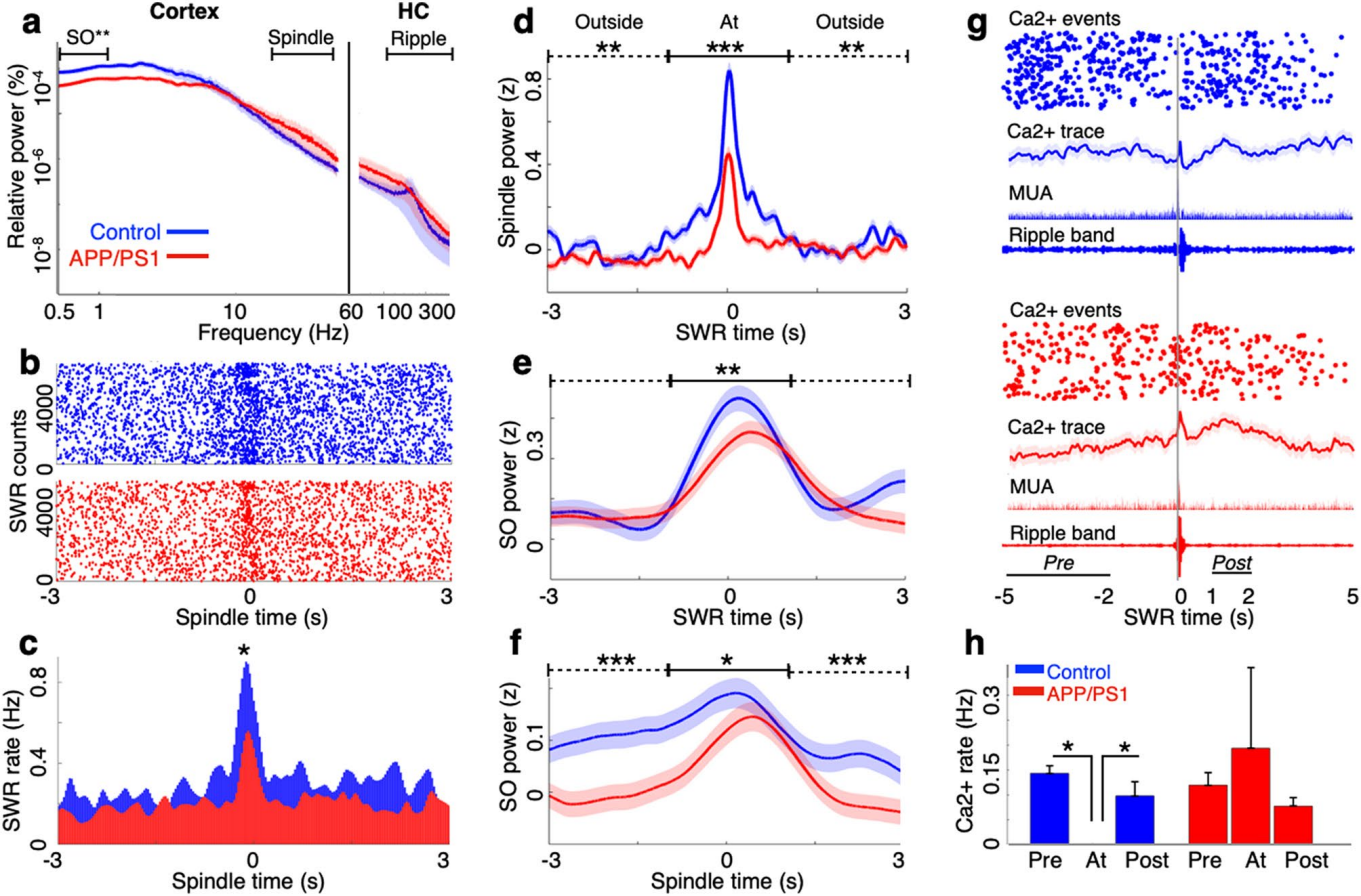
Aβ impairs cortical–hippocampal coordination and SWR modulation in slow wave sleep. (**a**) Cortical slow oscillation (SO) power in SWS was lower in APP/PS1 mice than in control mice, in contrast to comparable cortical spindle power and hippocampal ripple power. (**b**) Rasters and (**c**) peri-event time histograms (PETH) of SWRs around spindles in SWS showed reduced SWR-spindle co-occurrence in APP/PS1 mice compared to control mice; (**d**) spindle power was lower in APP/PS1 mice compared to control mice, at (solid line) and outside (dashed line) SWRs; (**e**) SO power was selectively reduced at SWRs in APP/PS1 compared to control mice; (**f**) SO power was lower in APP/PS1 compared to control mice, at and outside spindles. (**g**) SWR-triggered rasters of dynamic calcium events, averages of CA1 neuronal calcium traces, multi-unit activity (MUA) and ripple band of LFP in each genotype in SWS. (**h**) Across animals, SWR modulation of dynamic calcium activity observed in control mice was degraded in APP/PS1 mice. *p < 0.05; **p < 0.01; ***p < 0.001, ANOVA with post-hoc comparisons, 2-sided t-tests. control n = 6 mice, n = 3486 SWR events, n = 2984 spindle events; APP/PS1 n = 6 mice, n = 3214 SWR events, n = 2523 spindle events. Data in (**a**) and (**d, e, f**) are smoothed with a Gaussian window (σ = 200 ms and σ = 300 ms, respectively) for display purposes. Data are represented as mean ± S.E.M.

Cortical–hippocampal dialogue during sleep is critical in memory consolidation^16^ and is impaired in the triple transgenic 3xTg-mouse (Aβ and tau)^17^, as indicated by the dysfunction of physiological events and their temporal coupling. To isolate Aβ’s effects on cortical–hippocampal coordination, we focused on brain region-dependent events starting with the cortical SO, a brain rhythm that predominates in slow wave sleep. Consistent with prior observations^18,19^, we observed reduced cortical SO power in APP/PS1 mice (*p* = 0.0074, T = 3.34, *df* = 10; 2 sided t-test; Fig. 2a), while the power, rate, and duration of both thalamocortical spindles and hippocampal SWRs were comparable across the groups (spindle power: *p* = 0.5, T = 0.6, *df* = 10; SWR power: *p* = 0.6, T = − 0.4, *df* = 10; 2 sided t-test; Fig. 2a; Supplementary Table 3). To determine whether Aβ impaired the coordination of SWRs with spindles, we evaluated SWR events triggered on spindle onsets. A peak in the peri-event time histogram (PETH) was evident at spindle onset in both APP/PS1 and control mice, but the peak rate of SWRs at spindles was reduced in APP/PS1 mice (*p* = 0.03, T = 2.5, *df* = 9; 2-sided t-test; Fig. 2b, c). Similarly, the power of spindles triggered on SWRs was lower in APP/PS1 than control mice (at SWRs: *p* < 0.0001, T = 11.9, *df* = 2900; outside SWRs: *p* = 0.005, T = 2.7, *df* = 2900; 2-sided t-tests; Fig. 2d).

We next evaluated the coordination of cortical SOs and hippocampal SWRs. SWR-triggered SO power was reduced at SWRs in APP/PS1 mice (at SWRs: *p* = 0.003, T = 2.9, *df* = 2900; outside SWRs: *p* = 0.7; T = 0.3, *df* = 2900; 2-sided t-test; Fig. 2e). In contrast, spindle-triggered SO power was reduced in APP/PS1 mice both at spindle onset and outside of spindles (at spindles: *p* = 0.04, T = 2.0, *df* = 2952; outside spindles: *p* < 0.0001, T = 4.4, *df* = 2952; 2 sided t-test; Fig. 2f). Together, these results show that Aβ degrades the coordination of hippocampal SWRs with both the cortical SO and thalamocortical spindles.

Previous work has shown that hippocampal CA1 calcium events are dynamically suppressed at SWRs^15^. To determine whether Aβ affects this modulation, we evaluated calcium activity triggered by SWRs in SWS. Consistent with prior results^15^, control mice demonstrated a robust reduction in somatic calcium activity at SWR onsets, compared to baseline 2–5 s earlier and the immediate 1–2 s post-SWR period (repeated measures ANOVA: F_(2,9)_ = 15.4, *p* = 0.0012, post hoc comparison: Pre vs. At, *p* = 0.001; Post vs. At, *p* = 0.011; Fig. 2g, h). However, this modulation was diminished in APP/PS1 (repeated measures ANOVA: F_(2,9)_ = 15.4, *p* = 0.38; Fig. 2g, h). Similar results were observed when analyses were restricted to place cells (Control: repeated measures ANOVA: F_(2,9)_ = 6.5, *p* = 0.017, post hoc comparison: Pre vs. At, *p* = 0.04; Post vs. At, *p* = 0.02; APP/PS1: F_(2,9)_ = 0.5, *p* = 0.6; Supplementary Fig. S2). Thus, the modulation of neuronal calcium activity at SWRs was impaired in APP/PS1 mice.

### Aβ impairs hippocampal theta–gamma coupling and neuronal synchronization with the LFP in RUN and REM sleep

**Figure 3:**
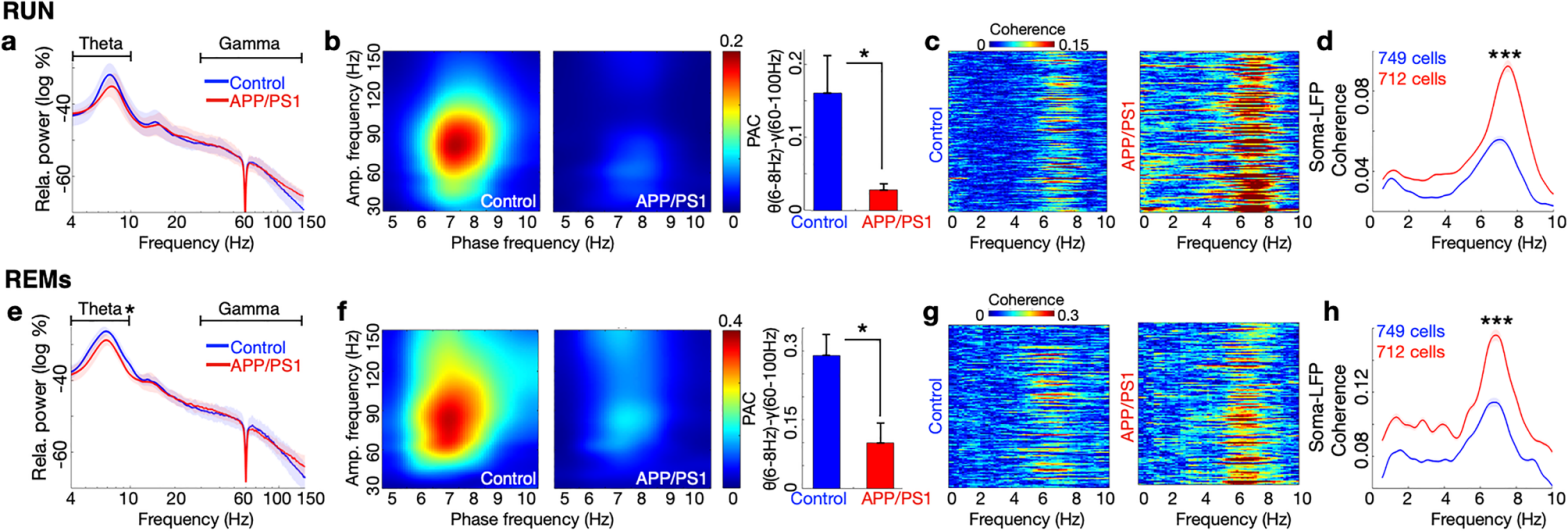
Aβ impairs hippocampal theta–gamma coupling in RUN and REM sleep and is associated with aberrant synchronization of neurons. (**a**) In RUN, LFP power in the theta and gamma bands in hippocampal CA1 were similar in control and APP/PS1 mice. (**b**) Phase-amplitude coupling (PAC) of theta (6–8 Hz)—gamma (60–100 Hz) was reduced in APP/PS1 mice compared to control mice. (**c**) Examples of coherence of each cell’s calcium trace with the LFP from one control mouse (146 cells) and one APP/PS1 mouse (124 cells) showed high coherence in the theta range during RUN. (**d**) Coherence of the neuronal calcium trace with the LFP in RUN was increased in the theta band in both control and APP/PS1 mice. This coherence was greater in APP/PS1 mice. (**e**) In REM sleep (REMs), LFP theta power was reduced in APP/PS1 mice compared to control, while gamma power was similar across the genotypes. (**f**), PAC of theta (6–8 Hz)–gamma (60–100 Hz) was reduced in APP/PS1 compared to control mice. (**g**) Coherence of each neuron’s calcium trace with the LFP in a control mouse (130 cells, left) and an APP/PS1 mouse (122 cells, right) showed high coherence in the theta range. (**h**) Coherence of the neuronal calcium trace with the LFP in REMs was increased in the theta band across genotypes and was greater in APP/PS1 than control mice. *p < 0.05; ***p < 0.001, 2-sided t-tests. Control n = 6 mice, APP/PS1 n = 7 mice. Data are smoothed with a Gaussian window (σ = 2 Hz) for display purposes. Data are represented as mean ± S.E.M.

Hippocampal theta–gamma phase amplitude coupling (PAC) has been linked to memory^20^ and has been reported to be compromised in awake behavior in an AD model expressing both Aβ and tau^21^. To determine whether Aβ deposits alone are sufficient to impair theta–gamma PAC, we evaluated theta–gamma PAC in the APP/PS1 model. In RUN, theta and gamma power were comparable across control and APP/PS1 mice (theta *p* = 0.3, T = 1.1, *df* = 10; gamma *p* = 0.8, T = 0.2, *df* = 10; 2 sided t-tests; Fig. 3a). Although both genotypes showed the highest PAC at the same range: theta phase at 6–8 Hz—gamma amplitude at 60–100 Hz, the extent of PAC was reduced in APP/PS1 compared to control mice (*p* = 0.028, T = 2.54, *df* = 10; 2-sided t-test; Fig. 3b). To determine individual neuron effects, we next examined the coherence of neuronal calcium traces with the LFP. A peak coherence in the theta range (~ 7–8 Hz) was observed in both control and APP/PS1 (Fig. 3c), indicating the synchronization of calcium fluctuations with the theta oscillation in RUN. Interestingly, this coherence was higher in APP/PS1 than control mice (*p* < 0.0001, T = 10.3, *df* = 1459; 2 sided t-test; Fig. 3d). Similar results of excessive synchronization were observed in APP/PS1 place cells (*p* < 0.0001, T = 5.2, *df* = 364; 2 sided t-test; Supplementary Fig. S3a). Of note, although behavior on the track was comparable between control and APP/PS1 mice (Supplementary Fig. S4a), across animals, significant correlations of theta-power and run velocity were diminished in APP/PS1 comparing to control mice (*p* = 0.02, T = 2.5, *df* = 11; 2 sided t-test; Supplementary Fig. S4b, c, d).

We next sought to determine whether these findings extended to REM sleep. In REM sleep, theta power was reduced in APP/PS1 mice compared to control mice, while gamma power remained unchanged (theta: *p* = 0.009, T = 3.2, *df* = 10; gamma: *p* = 0.8, T = 0.2, *df* = 10; 2-sided t-test; Fig. 3e). As in RUN, both genotypes showed the highest PAC at the same range: theta phase at 6–8 Hz—gamma amplitude at 60–100 Hz; and the extent of PAC was reduced in APP/PS1 mice compared to control mice (*p* = 0.013, T = 3.0, *df* = 10; 2-sided t-test; Fig. 3f). As in RUN, coherence of the calcium traces with the LFP was evident in both genotypes (Fig. 3g), reaching a maximum in the theta range (~ 7–8 Hz), and this coherence was higher in APP/PS1 than control mice (*p* < 0.0001, T = 10.4, *df* = 1459; 2 sided t-test; Fig. 3h). Similar results of excessive synchronization were observed in APP/PS1 place cells (*p* < 0.0001, T = 6.1, *df* = 364; 2 sided t-test; Supplementary Fig. S3b). Together, these results show that Aβ degrades theta–gamma PAC and alters neuronal synchronization with the LFP in both RUN and REM sleep.

### Acetylcholinesterase inhibitor-mediated rescue of Aβ-induced impairments are partial and behavior dependent

Basal forebrain cholinergic activity varies across the brain states of the sleep wake cycle, increasing in theta-associated states and falling in non-theta states^22,23^, and modulating the coordination of hippocampus related networks associated with successful memory updating^24,25^. As the basal forebrain cholinergic projection to the hippocampus degenerates in aged APP/PS1 mice^26,27^, recapitulating the prominent cholinergic impairment characteristic of AD^28–30^, we evaluated the possibility that rescue of this cholinergic impairment might reverse the aberrant physiology observed in APP/PS1 mice^31^.

**Figure 4:**
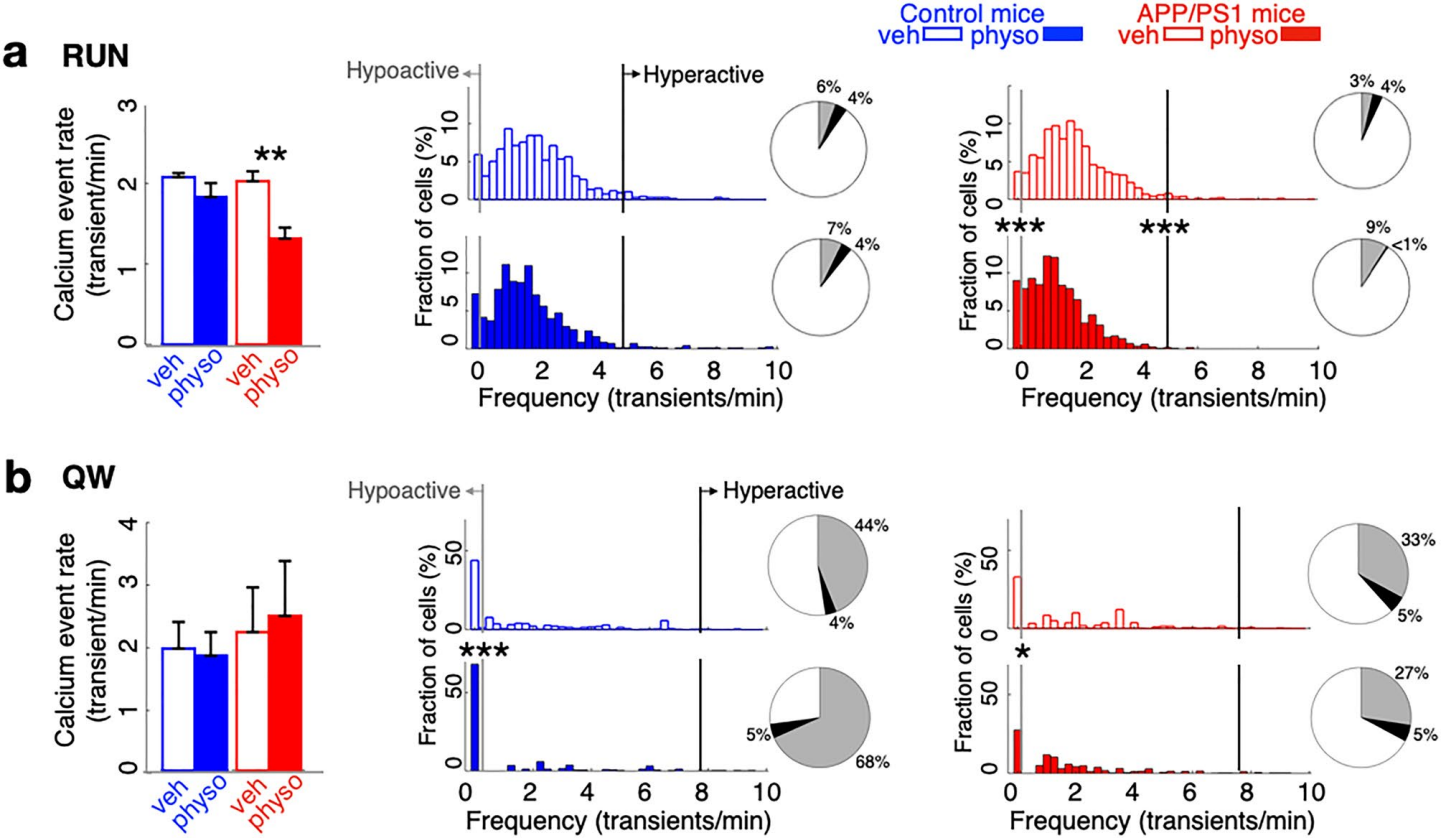
Acetylcholinesterase inhibitor-mediated rescue of Aβ-induced aberrant calcium activity is partial and behavior dependent. (**a**) Compared to treatment with vehicle, physostigmine injection (i.p.) reduced calcium event rates during RUN in APP/PS1 mice but not in control mice. In control mice, physostigmine had no effect on the proportion of hypoactive or hyperactive neurons. In APP/PS1 mice, physostigmine reduced the proportion of hyperactive neurons and increased the proportion of hypoactive neurons. (**b**) In quiet wakefulness (QW), physostigmine injection had no effect on calcium event rates compared to treatment with vehicle in APP/PS1 or control mice. In control mice, physostigmine increased the proportion of hypoactive cells. In contrast, in APP/PS1 mice physostigmine decreased the proportion of hypoactive cells. Grey: hypoactivity (< 0.25 transients/min); Black: hyperactivity (> 2 SD of mean calcium event rates under vehicle). *p < 0.05; **p < 0.01; ***p < 0.001, paired t-tests, χ^2^ tests; control n = 5 mice, n = 674 cells; APP/PS1 n = 5 mice, n = 1110 cells. Data are represented as mean ± S.E.M.

In RUN, treatment of control mice with the acetylcholinesterase inhibitor physostigmine modestly left shifted the overall distribution of calcium activity (distribution of calcium activity: *p* < 0.0001, K–S test) but had no effect on mean calcium event rates (rate: *p* = 0.1, T = 1.6, *df* = 5, paired t-test; hypoactive: *p* = 0.3, Chi = 0.9, χ^2^ test; hyperactive: *p* = 0.7, Chi = 0.08, χ^2^ test). In contrast, in APP/PS1 mice, physostigmine treatment reduced calcium event rates in RUN (*p* = 0.003, T = 4.1, *df* = 8, paired t-test) and markedly left-shifted the distribution of calcium activity (*p* < 0.0001, K–S test), increasing the proportion of hypoactive cells (Chi = 26.3, *p* < 0.0001, χ^2^ test) and decreasing the proportion of hyperactive cells (Chi = 23.8, *p* < 0.0001, χ^2^ test; Fig. 4a). Aβ-dependent effects of physostigmine on calcium activity were detected in both place cells and non-place cells (physostigmine vs. vehicle in APP/PS1 mice: place cells: *p* = 0.006, T = 3.6, *df* = 8; non-place cells: *p* = 0.0025, T = 4.3, *df* = 8; paired t-tests; Supplementary Fig. S5a). Notably, physostigmine at this dose had no effect on behavior including running speed in either group (Supplementary Table 4).

As basal forebrain cholinergic activity is higher in RUN than QW^32^, we hypothesized that the effect of physostigmine would be less pronounced in QW. Indeed, in QW, physostigmine had no effect on calcium event rates (physostigmine vs. vehicle: control *p* = 0.7, T = 0.3, *df* = 4; APP/PS1 *p* = 0.8, T = 0.2, *df* = 4; paired t-tests; Fig. 4b; Supplementary Fig. S5a) or the proportion of hyperactive cells in either group (control *p* = 0.4, Chi = 0.6; APP/PS1 *p* = 0.9, Chi = 0.01; χ^2^ tests). Even so, treatment with physostigmine increased the proportion of hypoactive cells in control mice (*p* < 0.0001, Chi = 60.8, χ^2^ test) but decreased the proportion of hypoactive cells in APP/PS1 (*p* = 0.01, Chi = 5.7, χ^2^ test) (Fig. 4b). Physostigmine treatment in APP/PS1 mice did not affect the proportion of place cells among aberrantly active cells in any behavioral state (Supplementary Table 5). Together, these results show that the effects of acetylcholinesterase inhibition are state- and Aβ-dependent, with physostigmine in the setting of Aβ expression selectively left-shifting calcium activity in RUN yet decreasing hypoactivity in QW.

**Figure 5:**
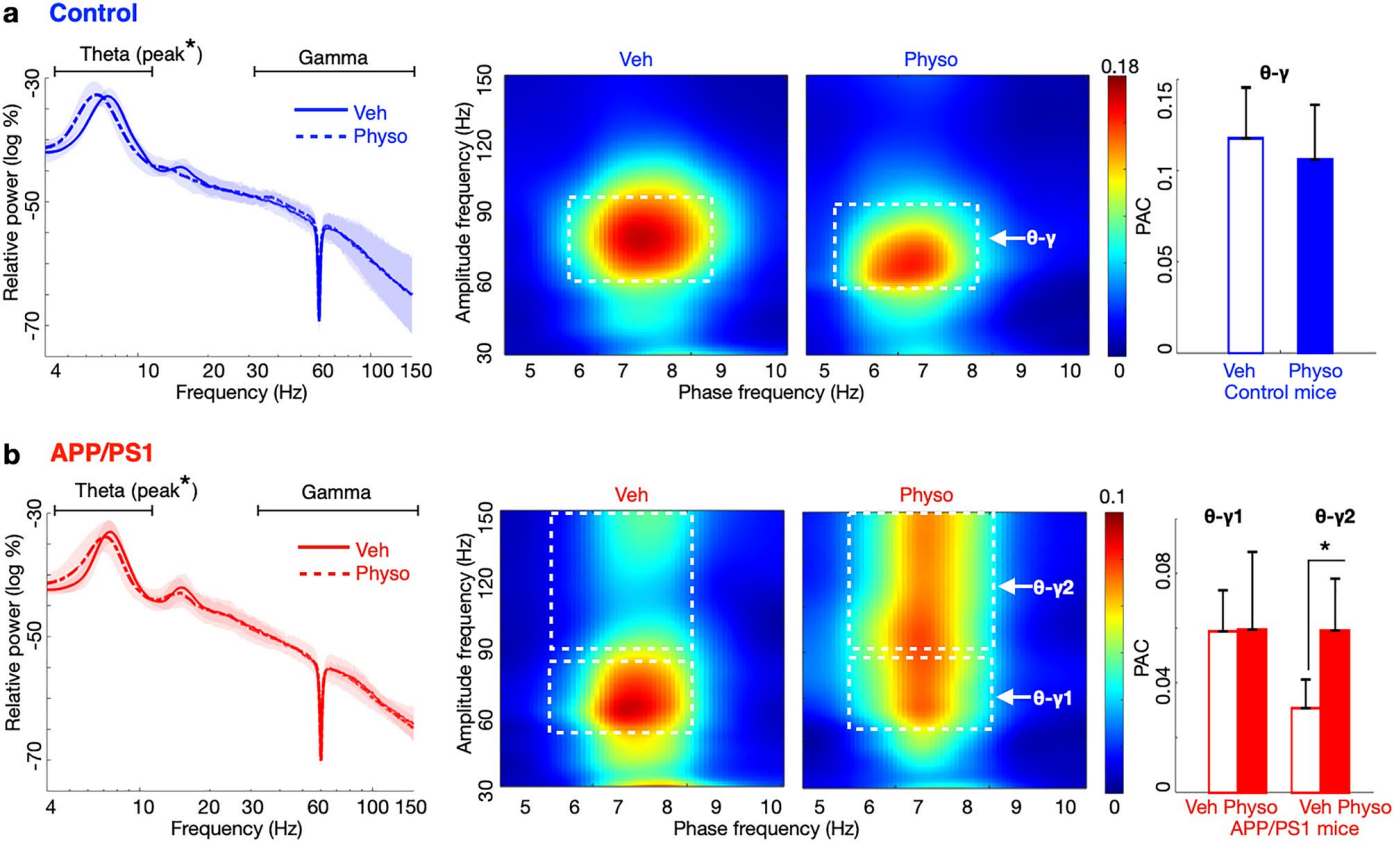
Acetylcholinesterase inhibitor treatment increased theta–gamma phase amplitude coupling in APP/PS1 mice. (**a**) In control mice, physostigmine treatment decreased the frequency of the theta band peak, without affecting power in theta and gamma bands or theta (phase 6–8 Hz)-gamma (amplitude 60–90 Hz) phase amplitude coupling (PAC). (**b**) In APP/PS1 mice, physostigmine treatment decreased the frequency of the theta band peak. Physostigmine had no effect on theta (phase 6–8 Hz)-gamma PAC in the 60–90 Hz range (θ–γ1) and selectively increased theta (phase 6–8 Hz)-gamma PAC in the 90–150 Hz range (θ–γ2). White dashed squares: regions of quantified PAC; *p < 0.05; paired t-tests. Control n = 7, APP/PS1 n = 7 mice. Data in (**a**) left and (**b**) left are smoothed with a Gaussian window (σ = 2 Hz) for display. Data are represented as mean ± S.E.M.

Given the distinct effects of acetylcholinesterase inhibition in APP/PS1 and control mice, we next examined its effects on hippocampal LFP oscillations. Although physostigmine treatment had no effects on theta and gamma power in either genotype (control theta: *p* = 0.3, T = 1.0, *df* = 5; control gamma: *p* = 0.4, T = 0.7, *df* = 5; APP/PS1 theta: *p* = 0.1, T = 1.6, *df* = 5; APP/PS1 gamma: *p* = 0.8, T = 0.1, *df* = 5; paired t-tests), it decreased the frequency of the theta band peak in both (control theta peak decreased from 7.4 ± 0.06 Hz to 6.8 ± 0.1 Hz, *p* = 0.01, T = 3.7, *df* = 5; APP/PS1 theta peak decreased from 7.5 ± 0.09 Hz to 6.9 ± 0.2 Hz, *p* = 0.04, T = 2.6, *df* = 5; paired t-tests) (Fig. 5a, b left). Although theta phase 6–8 Hz and gamma amplitude 60–90 Hz showed high PAC in both genotypes with or without physostigmine treatment (control: *p* = 0.3, T = 1.0, *df* = 5; APP/PS1: *p* = 0.7, T = 0.3, *df* = 4; paired t-tests), in APP/PS1 mice physostigmine selectively increased the gamma range that coupled with theta phase to 90–150 Hz (*p* = 0.04, T = 2.9, *df* = 4; paired t-test) (Fig. 5a, b). Together, these results show that physostigmine’s partial rescue of aberrant neuronal activity in APP/PS1 mice was associated with an increase in the range of gamma that coupled with theta phase.

As a final assessment, we evaluated the hippocampal LFP for evidence of epileptic activity. Consistent with prior results^33^, epileptic-like spikes in the hippocampal LFP were present at low frequency in 6/7 APP/PS1 mice when evaluated across all behavioral states. Epileptic spikes were most frequent in SWS and REM sleep (SWS: control: 0; APP/PS1: 0.0073 ± 0.0033 Hz; *p* = 0.032, 1-sided t-test), providing a nonspecific electrophysiologic marker of hippocampal dysfunction that may contribute to the perturbations of dynamic calcium activity observed here.

## Discussion

We combined large-scale calcium imaging of hippocampal CA1 neurons with simultaneous LFP recordings in a mouse model of AD to evaluate Aβ’s effects on neuronal activity across the sleep–wake cycle. We found that Aβ’s effects on hippocampal neuronal circuit function were profoundly dependent on brain states associated with both memory encoding and consolidation. Aβ drove hyperactivity during RUN and SWS, yet suppressed activity in QW and REM sleep. Treatment of APP/PS1 mice with an acetylcholinesterase inhibitor rescued these aberrant activities in RUN and QW, but also suppressed RUN-associated calcium activity.

Previous observations of the effects of Aβ deposits on calcium activity were conducted under anesthesia or immobility with light anesthesia^2–4^, and showed that Aβ broadened the distribution of activity, increasing the proportions of both inactive and hyperactive cells^2–4^. However, anesthesia brings complexity, altering neurophysiology, calcium handling, and vascular, respiratory and thermal regulation^34–36^. Indeed, the behavioral state-associated LFP oscillations that entrain neuronal activity in the hippocampus, including hippocampal theta associated with RUN and REM sleep and implicated in memory encoding^11^, and the prominent irregular activity and SWRs associated with QW and SWS that are implicated in memory consolidation^12^, are sensitive to anesthetics such as isoflurane^37^. In this context, the reduction of fMRI BOLD signal^38^ and glucose metabolism^39^ observed in imaged, resting patients with AD has long been difficult to reconcile with neuronal hyperactivity observed in AD mouse models under anesthesia. In contrast, the BOLD reduction in imaged Alzheimer’s patients would seem to align well with the reduction of dynamic calcium activity that we observed in QW in APP/PS1 mice, although additional explanations such as concomitant tau pathology^4,40^ may also be contributory.

The mechanistic understanding of how AD causes progressive brain dysfunction is still limited. A recent study of calcium activity in young (4–5 month) APP/PS1 mice, at an age when soluble Aβ levels are elevated but prior to significant hippocampal Aβ deposition or memory impairments, showed that hippocampal network firing stability was normal in waking states but was disrupted in NREM and anesthesia states^41^. In RUN behavior, no evidence of hippocampal hyperactivity was observed, while the homeostatic down-regulation of CA1 firing state was disrupted during sleep, and general anesthesia in several familial AD mouse models resulted in network hyperactivity^2,42,43^. Together with the present findings acquired in old APP/PS1 mice that harbor hippocampal Aβ deposits, these observations suggest that RUN-associated hippocampal neuronal hyperactivity develops with plaque deposition and may reflect a physiological correlate of memory impairment. The present findings are also supported by recent work in young 3 × Tg-AD mice showing higher neural ensemble calcium activity in CA1 and impaired spatial coding at an age when Aβ deposits appear in the CA1 pyramidal cell layer while tau pathology is not robustly observed^44^. Considering the critical role of the sleep–wake cycle in hippocampal function^11,12^, Aβ deposit-associated aberrant activity in other behavioral states including QW, SWS, and REM sleep may also contribute to memory impairment in AD. These findings highlight the value of understanding Aβ’s effects across brain states.

The role of hippocampal theta–gamma phase amplitude coupling (PAC) in memory performance and its impairment in AD models have suggested that altered theta–gamma PAC may serve as a biomarker of AD^45^. Indeed, PAC of hippocampal theta with cortical or hippocampal gamma in Aβ and tau models has been the focus of many in vitro^46^, anesthesia^47,48^, and freely moving^21,49–51^ studies. Consistent with this possibility, we found in aged APP/PS1 mice harboring deposits of Aβ, when hippocampal-dependent memory impairments are evident^14,52,53^, that although hippocampal theta power was preserved in RUN and reduced in REM sleep, PAC of hippocampal theta with hippocampal gamma was reduced in both states. The impairment in hippocampal theta–gamma PAC arose in association with aberrantly increased theta-range coherence of neuronal calcium fluctuations with the LFP, that may reflect a breakdown of local cell assembly organization and dynamics^54^. Together with prior studies showing that soluble Aβ has no effect on PAC of hippocampal theta with hippocampal gamma in either RUN^50^ or REM sleep^50,54^, while Aβ deposition is associated with reduced PAC in RUN^49^, these results suggest that the reduction of theta–gamma PAC in REM sleep may require Aβ deposition. In this context, it is interesting to note that deletion of the amyloid precursor protein (APP) that generates Aβ has also been found to degrade theta–gamma PAC^55^, suggesting that physiological levels of APP’s enzymatic products contribute to theta–gamma PAC under normal conditions. Theta–gamma PAC in RUN has been implicated in working and episodic memory^10^ as well as in long term spatial memory retrieval^56^. However, the function of theta–gamma PAC in REM sleep remains unclear. It is possible that it contributes to REM sleep-dependent contextual memory consolidation^57^ and to the REM sleep replay of spatial experience^58^, which may depend upon and be reflected in correlations in theta–gamma PAC across these states. If so, Aβ-mediated impairments in theta–gamma PAC in REM sleep may also contribute to impairment of hippocampal-dependent memory in AD.

Interestingly, Aβ’s effects in SWS were distinct from its effects in RUN and REM sleep, as it increased the proportion of hyperactive neurons, altered the coordination of hippocampal neuronal calcium activity at SWRs, and degraded both cortical SO power and hippocampal-cortical coordination. It is intriguing that cortical SO power was preferentially reduced at hippocampal SWRs, in line with the temporal association between these two oscillations in SWS^59^. This observation suggests that the Aβ-associated reduction in cortical SO power may reflect a failure of cortical–hippocampal coordination that also manifested in the prominent impairment of coordination between thalamocortical spindles and hippocampal SWRs. Modulation of hippocampal activity by a neocortical DOWN state that precedes SWR events has been suggested to contribute to the suppression of calcium transients immediately prior to SWRs^60–63^. Disruption of this coordination may contribute to the degraded coordination of calcium activity at SWRs. Sleep spindle-SWR coordination has been suggested to play a critical role in memory consolidation, providing a mechanism by which replayed activity in the hippocampus can be transferred to cortex^60,64^. As a reduction in spindle-SWR coordination has been previously reported in APP/PS1 mice prior to Aβ deposition^54^, when memory is still normal, this effect is likely to be mediated by soluble Aβ. Thus, as Aβ accumulates, soluble Aβ may first impair SO-associated thalamocortical spindle-hippocampal SWR coordination, and only later in association with Aβ deposition and impairment of hippocampal theta–gamma PAC, is memory impaired. In addition, these results show that Aβ alone is sufficient for hippocampal-neocortical disconnection^65,66^, even without the accompanying neurofibrillary tangles and neuronal loss that characterize AD. Aβ’s effects on network function may also contribute to the disruption of the sleep–wake cycle that patients with AD experience^67^ and to impaired SWS-dependent clearance of neurotoxic metabolic products including Aβ^68–70^.

Although other therapeutic strategies for AD have received more attention in recent work, acetylcholinesterase inhibitors are still the mainstay of treatment for AD, modestly but significantly improving memory in early to moderate disease^71,72^. In normal animals, activating basal forebrain cholinergic cells with optogenetic or chemogenetic strategies has been shown to increase calcium activity and reduce sharp wave ripples^73,74^. However, the effects of acetylcholinesterase inhibitors on hippocampal systems level physiology in AD models has not been systematically explored. Here, using an AD model that recapitulates not only Aβ deposition but also progressive cholinergic dysfunction^26^, we found that the short acting acetylcholinesterase inhibitor physostigmine partially rescued both dynamic calcium activity and theta–gamma PAC.

Although acetylcholinesterase inhibitors like physostigmine are neurotoxic and impair behavior at high dose^75^, physostigmine had no effect on locomotor activity at the dose studied, consistent with prior results^76,77^. In RUN, physostigmine left-shifted calcium activity in APP/PS1 mice, thereby reducing hyperactivity, but, in so doing, it increased the proportion of hypoactive cells, an effect which may limit the clinical benefits of acetylcholinesterase inhibitors. In addition, physostigmine improved theta–gamma PAC in APP/PS1 by expanding PAC from gamma 60–90 Hz to involve a higher gamma range (90–150 Hz; also referred to as the epsilon band^78^), while also reducing the peak frequency of hippocampal theta. Behavior-dependent brain states across the sleep–wake cycle determine the LFP characteristics and calcium dynamics of CA1 as well as basal forebrain cholinergic activity^11^. The distinct effects of acetylcholinesterase inhibitor treatment on RUN- and QW-associated aberrant calcium activity of APP/PS1 mice suggest that the therapeutic actions of acetylcholinesterase inhibitors in AD may require theta-dependent brain states (i.e., RUN and REM sleep) associated with high cholinergic activity, and we anticipate that physostigmine’s actions reflected in the partial rescue of both hippocampal neuronal activity and theta–gamma PAC are likely to contribute to the therapeutic benefit of acetylcholinesterase inhibitors in AD. Together, these observations identify putative network level effects of acetylcholinesterase inhibitors and also highlight the need for novel therapeutic strategies to target and reverse Aβ-associated network impairments that did not respond to acetylcholinesterase inhibitor treatment.

It should be noted that the human AD condition is, of course, more complicated than mouse models. For example, the presence of tau inclusions may well impact neural systems as well as Aβ deposits, and this synergy is important to understand and model^4,40^. In addition to these and other challenges of translation from mouse models to human disease, however, our current work highlights the critical effects of state dependent neural system biology on AD-related phenomena, and suggest strongly that both animal models of disease and design of human clinical trials need to potentially be reevaluated with differential effects during wakefulness and sleep considered as central to understanding neural system directed therapeutics.

Strengths of this study include the evaluation of hippocampal circuit function across the sleep- wake cycle in a freely behaving model of AD, at an age when this model manifests both amyloid plaques and well-described and robust hippocampal-dependent memory impairments^14,52,53^. Another strength is the integration of hippocampal calcium imaging together with electrophysiological recordings, which enhances understanding of calcium activity in relation to LFP and as a function of brain state. This paradigm provides an approach that in future work can be used to decode the linkage between a single neuron’s activity and network organization during different memory states. Limitations include the use of a short acting acetylcholinesterase inhibitor, which precluded assessment of drug effects during sleep, although effects of this agent on both calcium activity and LFP were readily detected in the waking states evaluated. While this study has focused on the pathophysiological consequences of Aβ pathology in plaque bearing mice, in future work, it will be worth considering the critical time window for network dysfunction that arises during progression of AD using similar approaches. Although the APP/PS1 mouse is a robust and well-studied model of amyloidosis, APP intracellular domain (AICD) has recently emerged as an APP-related peptide that can enhance the after-hyperpolarization, weaken neuron firing in the gamma frequency range, and reduce gamma^79^. Although AICD is believed to be rapidly degraded^80–82^ and we did not observe a reduction in gamma power in the APP/PS1 model, it remains possible that the AICD peptide or a C terminal fragment such as β-CTF^83^ contributed to some APP/PS1 associated observations. Future work exploring this possibility will be worthwhile. Even so, many reports have shown that the direct application of Aβ oligomers in vivo recapitulates features observed in the APP/PS1 model^2,84–86^, inducing neuronal calcium hyperactivity in wild-type mice^87^ and disrupting hippocampal oscillations and memory dependent plasticity. Together, these findings suggest that Aβ is the primary driver of pathophysiology in the APP/PS1 model.

In summary, the present findings in an established mouse model of AD identify multiple distinct effects of Aβ on neural circuit function that vary across behavioral brain states. Aβ not only degraded hippocampal neuronal activity but also disrupted intra-hippocampal and hippocampal-neocortical coordination that contribute to encoding and consolidation phases of memory formation. Accounting for the state-dependent effects of Aβ is likely to improve preclinical drug selection for clinical trials in AD.

## Materials and methods

All procedures were approved by the Institutional Animal Care and Use Committees of the Massachusetts General Hospital and followed the ethical guidelines of the US National Institutes of Health. *ARRIVE guidelines*: This study is reported in accordance with ARRIVE guidelines (Animal Research: Reporting of in vivo Experiments; https://arriveguidelines.org).

### Surgical preparation

All procedures were similar to our recent study^15^. B6C3-Tg (APPswe, PSEN1dE9) 85Dbo/Mmjax transgenic mice (APP/PS-1) and their non-transgenic control littermates were injected with 1 µL AAV-hSyn-GCaMP6f-GFP (titer 8 × 10^12^ mL^−1^) into the hippocampal CA1 region (anterior–posterior (AP) −2.1 mm, medial–lateral (ML)  −1.65 mm, dorsal–ventral (DV)  −1.4 mm from Bregma) at the speed of 0.2 µL/min under anesthesia (isoflurane 1.5–2%), and then underwent grin lens implantation (1 mm diameter) above CA1 (DV −1.2 mm from dura) 2 weeks later. A designed drivable probe containing 7 independent stereotrodes with different lengths (99.95% HML insulated tungsten wire, 20 µm diameter, California Fine Wire Company) was implanted ipsilaterally to sample cortical and hippocampal activity in proximity to the lens (AP −2.8 mm, ML −2.6 mm from Bregma, −36° relative to the AP axis, −39° relative to the inter-aural axis; DV −1.2 mm from dura), with cerebellar ground. An EMG wire was placed in the posterior neck musculature in a subset of mice. The microscope baseplate was attached 3 weeks later. Carprofen (10 mg/kg, s.c) was used for postoperative analgesia. The stereotrodes were slowly lowered to sample cortex, the CA1 pyramidal cell layer and stratum radiatum. The accuracy of GCaMP6f expression and lens and probe positioning were confirmed with postmortem evaluation and histology. Animals were housed in individual cages after virus injection and received 3-days handling before recording.

### Calcium imaging, electrophysiology, and behavior

The Inscopix™ mini-microscope and acquisition system were used for calcium imaging with sampling rate of 20 fps (50 ms exposure time). Local field potential (LFP) was acquired at 2 kHz, 0.5–900 Hz filtering from each contact site on the probe (Neuralynx™). Head position and direction were monitored with overhead camera tracking of two LED diodes mounted on the headstage. Before recording, animals were trained to run on a linear track (200 cm) between two goal locations until they could run at least 10 laps in each 30-min training session (3 trials, 10 min/trial). Electrophysiological recordings were acquired throughout behavioral sessions. Calcium imaging was acquired simultaneously in 10 min blocks, with 4–5 min intervals off-camera to avoid photobleaching. Quiet wakefulness (QW), slow-wave sleep (SWS), and REM sleep were acquired immediately after track sessions in the home cage (replaced with a sleep chamber in pharmacological experiments) within the same recording room. Animals were housed in individual cages with a 12 h light/12 h dark- standard light cycle. Exploratory behavior (RUN) was defined as running behavior (> 3 cm/s) on the track. QW was classified during periods of wakeful immobility (< 0.5 cm/s, > 10 s, with eyes open), both prior to sleep and following arousal events and postural changes. SWS was classified by irregular activity and SWRs arising in the setting of prolonged immobility (< 0.5 cm/s for at least 3 min) in a sleep posture with delta/theta LFP power ratio > 2. REM sleep was classified by theta/delta LFP power ratio > 2 arising out of SWS, with each epoch of REM sleep lasting at least 30 s. Where available, EMG data confirmed designations of state.

### Pharmacological experiments

For pharmacological experiments, calcium imaging and electrophysiology recordings in RUN and QW were acquired 10–60 min after injection of vehicle (saline, i.p., 10 ml/kg). Animals then underwent treatment with physostigmine (0.25 mg/kg, i.p), and data were acquired from 10 to 60 min post-injection^77^. Sleep was not examined under physostigmine given the short half-life of the drug.

### Data analysis

All analyses were performed using MATLAB (2019b).

#### Stereotrode selection

Local field potentials across the stereotrodes were reviewed and filtered to obtain hippocampal SWRs (Blackman filter; 100–300 Hz), theta (4–10 Hz), and delta oscillations (0.5–4 Hz), gamma (30–150 Hz), as well as cortical spindles (12–16 Hz) and SOs (0.5–1 Hz). The spectrogram of each channel of each stereotrode was calculated using the Chronux toolbox^88^ with a moving window of size 2 s and step size 10 ms. A stereotrode in s. radiatum characterized by high amplitude theta below the cell layer (and without SWRs), was used for analysis of theta and gamma oscillations in RUN and REM sleep; a stereotrode in s. pyramidale characterized by high amplitude SWRs (100–300 Hz) and unit activity was used for analysis of SWRs in QW and SWS; a stereotrode in cortex characterized by slow oscillations and sleep spindles was used to measure these features in SWS. Analyses of SWS were conducted using a cerebellar reference. A local reference above the hippocampus was used for the remaining analyses to reduce mechanical artifacts.

#### Calcium imaging

Established software was used for initial processing of calcium activity (Mosaic, Inscopix, Palo Alto, California). This includes calcium data registration, using rigid-body motion correction as required, followed by PCA/ICA analysis of ΔF/F datasets, as described previously^89^. ICs were reviewed manually to derive ΔF/F time series for each soma. Calcium events were identified as threshold crossings of IC traces, with threshold defined as 2 Z scores above the mean. The onset of each event was defined as the time at which the event reached 30% of its maximum amplitude. Hypoactivity was defined as < 0.25 transients/min^90^. Hyperactivity in each behavioral state was defined relative to calcium activity in control mice, using a threshold of 2 SD above the mean. For analyses of calcium event rates, data were analyzed at the animal level; for analyses of hypo-/hyperactivity, cells of each genotype were aggregated across animals to account for the low frequency of aberrant activity.

#### LFP power spectra and phase-amplitude-coupling

LFP power spectra were computed from 0 to 200 Hz using the Chronux toolbox^88^. Quantification of power was conducted using the sum of relative power of each frequency band: theta 4–10 Hz^11^, gamma 30–150 Hz^91^. Cross-frequency coherence (CFC) phase-amplitude-coupling (PAC) was calculated with 1–25 Hz for phase frequency and 1–300 Hz for amplitude frequency using the MATLAB toolbox https://data.mrc.ox.ac.uk/data-set/matlab-toolbox-estimating-phase-amplitude-coupling^92,93^. The CFC measure calculates the coherence between the time-varying energy of gamma and the unfiltered raw signal believed to contain the modulating frequency^92^, Quantification of PAC was calculated as the mean value of PAC in the region of high coupling^54^.

#### Coherence of calcium trace and LFP in RUN and REM sleep

LFPs were downsampled to 20 Hz to match the calcium imaging sampling rate. Coherence of calcium traces with LFP was calculated using the Chronux toolbox^88^. Quantification of coherence was calculated using the maximum coherence in the theta range.

#### SO, spindle and SWR in SWS

Spindle and SWR detection was based on^94^: LFP was bandpass filtered at 12–16 Hz for spindles and at 100–300 Hz for SWRs and Hilbert transformed. Spindles were detected using an amplitude threshold of 1.25 Z score with minimum length of 200 ms and visually reviewed. SWRs were detected using an amplitude threshold of 3 Z score with minimum length of 20 ms and visually reviewed. Spindle and SWR onsets were defined as the time when the signal first crossed threshold. Rasters and peri-event time histograms (PETHs) of SWR-spindle co-occurrence were calculated with 30 ms bin size; LFP power triggered on SWR or spindle onsets was calculated with 1 ms bin size; calcium traces triggered on SWR or spindle onsets were calculated with 50 ms bin size.

#### Place cell detection

Spatial tuning curves (3 cm bins) were constructed for each cell for each running direction using all detected calcium events acquired during running (> 3 cm/s). Spatial information was computed for each spatial tuning curve and compared to spatial information distributions of 1000 shuffled versions of the data to test significance (Monte Carlo *p* value < 0.05)^15,95^. In each shuffle, calcium events were reassigned to random times and spatial information was recomputed.

### Histology

Mice were euthanized with Fatal Plus (100 mg/kg, i.p.) and perfused with 4% paraformaldehyde solution. Brain sections were obtained by microtome (50 µm-thickness). Fluorescence expression and electrode location were evaluated with microscopy (Zeiss Axio Imager Z2). Expression of amyloid-β plaque was assessed using amyloid-β primary antibody (IBL 18584, 1:500) and secondary fluorescent antibody (Invitrogen A21207, 1:500).

### Statistical analysis

All group values were represented as mean ± S.E.M. Student’s *t*-test and one-way ANOVA with repeated measures followed by post hoc tests were used for statistical analyses according to the experimental design (SPSS). (All statistics are reported in the statistical source file). The significance threshold was set at *p* < 0.05.

## Supplementary Information

Supplementary Information.

## Data Availability

The datasets generated during and/or analyzed during the current study are available in the Dryad repository, https://datadryad.org/stash/share/L4kBWcOvzVh7vzoZizyrm2D6vazcSC27wxSm2KahmYo.
