# Supplementary material for: Disruption of hippocampal neuronal circuit function depends upon behavioral state in the APP/PS1 mouse model of Alzheimer’s disease
Source: Sci Rep. 2022 Dec 5;12:21022. doi: 10.1038/s41598-022-25364-2 (PMC9723144; doi:10.1038/s41598-022-25364-2)
Supplement: Supplementary file 1 — Supplementary Information. [file 41598_2022_25364_MOESM1_ESM.pdf]

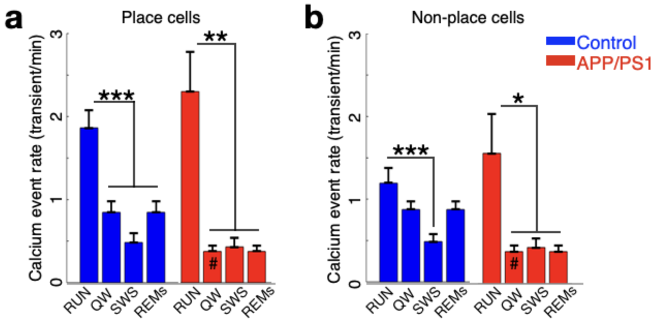

Fig. S1: Calcium event rates in place cells and non-place cells. a, In control and APP/PS1 mice, calcium event rates of place cells were higher in RUN than all other states. In direct comparisons, place cells in APP/PS1 mice had lower calcium event rates than control mice in QW (#). **b,** In control mice, calcium event rates of non-place cells were higher in RUN than SWS. In APP/PS1 mice, calcium event rates of non-place cells were higher in RUN than all other states. In direct comparisons, non-place cells in APP/PS1 mice had lower calcium event rates than control mice in QW (#). Mean calcium event rate in each mouse is shown.). *, # $p<0.05$, ** $p<0.01$, *** $p<0.001$; ANOVA with post-hoc comparisons, 2-sided t-tests. Control=6 mice, 192 place cells, 557 non-place cells; APP/PS1=7 mice, 174 place cells, 538 non-place cells. Data in bar graphs are represented as mean ± s.e.m.

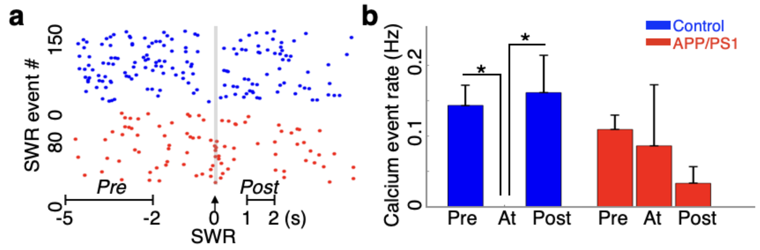

Fig. S2: Aβ impairs SWR modulation of place cell calcium activity in slow wave sleep. a, Rasters of place cell dynamic calcium events showed reduced activity at SWRs in a control mouse but not in an APP/PS1 mouse. **b,** Across animals, SWR modulation of place cell activity observed in control mice was degraded in APP/PS1 mice. * $p<0.05$, ANOVA with post-hoc comparisons. Data in are represented as mean ± s.e.m.

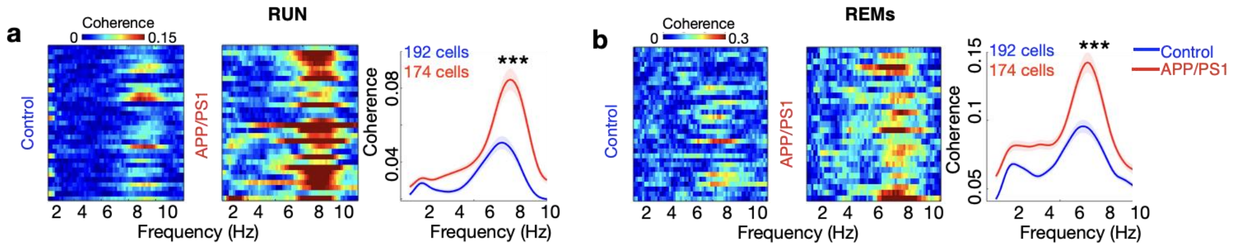

Fig S3: Aβ is associated with aberrant synchronization of place cells in RUN and REM sleep. **a**, In RUN, examples of coherence of place cell calcium traces with the LFP from one control mouse (33 cells) and one APP/PS1 mouse (30 cells) showed high coherence in the theta range. This coherence was increased in the theta band in both genotypes but was greater in APP/PS1 mice. **b**, In REM sleep (REMs), examples of coherence of place cell calcium traces with the LFP from one control mouse (32 cells) and one APP/PS1 mouse (27 cells) showed high coherence in the theta range. This coherence was increased in the theta band in both genotypes but was greater in APP/PS1 mice. *** $p < 0.001$, 2-sided t-tests. Control n=6 mice, n=192 place cells; APP/PS1 n=7 mice, n=174 place cells. Data are smoothed with a Gaussian window ($\sigma = 2$ Hz) for display purposes. Data are represented as mean ± s.e.m.

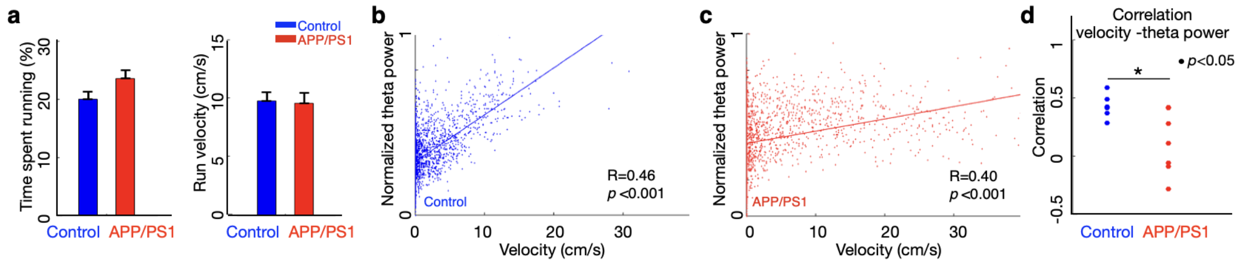

Fig. S4: Behavior on the track and association of running velocity with hippocampal theta. **a,** Time spent running (velocity > 3 cm/s) and mean run velocity were comparable across control and APP/PS1 mice. **b**, Correlations of mean theta power and run velocity in example control and **c**, APP/PS1 mice. **d**, Across animals, significant correlations of theta power and run velocity were higher in control mice than APP/PS1 mice ($p=0.02$, $T=2.5$, $df=11$, 2 sided t-test).

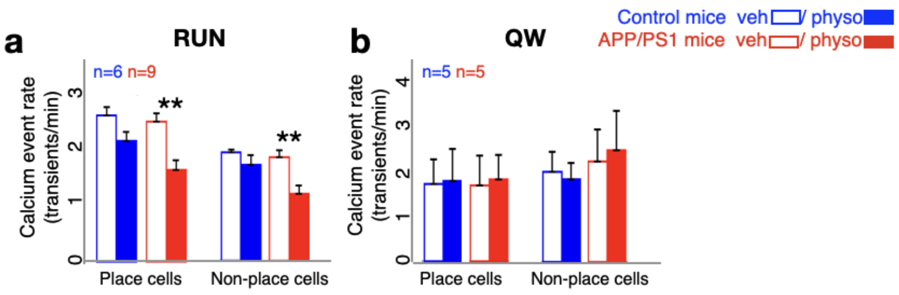

Fig S5: Effects of acetylcholinesterase inhibitor treatment in place cells and non-place cells depend upon Aβ and behavioral state. a, In RUN, physostigmine reduced calcium event rates of place cells and non-place cells in APP/PS1 mice but not in control mice (physostigmine vs. vehicle: control place cells: p=0.1, T=1.9, df=5; control non-place cells: p=0.1, T=1.5, df=5; APP/PS1 place cells: p = 0.006, T = 3.6, df = 8; APP/PS1 non-place cells: p = 0.0025, T = 4.3, df = 8; paired t-tests). **b**, In QW, physostigmine had no effect on calcium event rates of place cells and non-place cells of either genotype (physostigmine vs. vehicle: control place cells: p=0.9, T=0.1, df=4; control non-place cells: p=0.6, T=0.5, df=4; APP/PS1 place cells: p = 0.9, T =0.1, df = 4; APP/PS1 non-place cells: p =0.8, T = 0.2, df = 4; paired t-tests). ** p<0.01; Data are represented as mean ± s.e.m.

Supplementary tables

Table 1: The proportion of aberrantly active cells that are place cells in each behavioral state in APP/PS1 mice

	RUN	QW	SWS	REMs
Hypoactive	4%	24%	23%	22%
Hyperactive	30%	25%	27%	30%

Table 2: Behavioral characterization

	On track	Spontaneous awake			
	Speed (cm/s)	Moving (%)	Exploring (%)	Resting (%)	Grooming (%)
Control	11.1 ± 1.5	61.4 ± 5.0	32.0 ± 3.0	1.7 ± 0.4	3.2 ± 0.6
APP/PS1	10.7 ± 1.0	62.0 ± 5.4	34.9 ± 4.0	2.2 ± 0.9	4.0 ± 0.3
	T=0.2, p=0.8	T=0.09, p=0.9	T=0.5, p=0.6	T=0.4, p=0.7	T=1.1, p=0.3

Table 3: Thalamocortical spindle and hippocampal SWR rates and durations in SWS

	Spindle rate (Hz)	Spindle duration (s)	SWR rate (Hz)	SWR duration (s)
Control	0.1 ± 0.008	0.2 ± 0.001	0.1 ± 0.04	0.02 ± 0.0001
APP/PS1	0.1 ± 0.006	0.2 ± 0.002	0.1 ± 0.03	0.02 ± 0.0001
	T=0.2, p=0.8	T=1.1, p=0.2	T=0.1, p=0.9	T=0.01, p=0.9

Table 4: Run speed with acetylcholinesterase inhibitor treatment

	Vehicle (cm/s)	Physostigmine (cm/s)
Control	9.8 ± 6.5	7.6 ± 4.8
APP/PS1	8.6 ± 5.3	7.9 ± 4.8
	T=1.2, p=0.3	T=1.4, p=0.2

Table 5: The effect of physostigmine treatment on the proportion of place cells among aberrantly active cells

	RUN Hypoactive	RUN Hyperactive	QW Hypoactive	QW Hyperactive
Vehicle	2%	19%	12%	2%
Physostigmine	7%	20%	16%	4%
	Chi=1.1, p=0.2	Chi=8.6e-04, p=0.9	Chi=1.7, p=0.1	Chi=0.3, p=0.5
